# Supplementary material for: Anti-Human CD9 Fab Fragment Antibody Blocks the Extracellular Vesicle-Mediated Increase in Malignancy of Colon Cancer Cells
Source: Cells. 2022 Aug 10;11(16):2474. doi: 10.3390/cells11162474 (PMC9406449; doi:10.3390/cells11162474)
Supplement: Supplementary file 1 [file cells-11-02474-s001.zip › cells-1832743-supplementary.pdf]

cells MDPI

Anti-Human CD9 Fab Fragment Antibody Blocks the Extracellular Vesicle-Mediated Increase in Malignancy of Colon Cancer Cells

Mark F. Santos, Germana Rappa, Simona Fontana, Jana Karbanová, Feryal Aalam, Derek Tai, Zhiyin Li, Marzia Pucci, Riccardo Alessandro, Chikao Morimoto, Denis Corbeil and Aurelio Lorico

Supplementary Figures

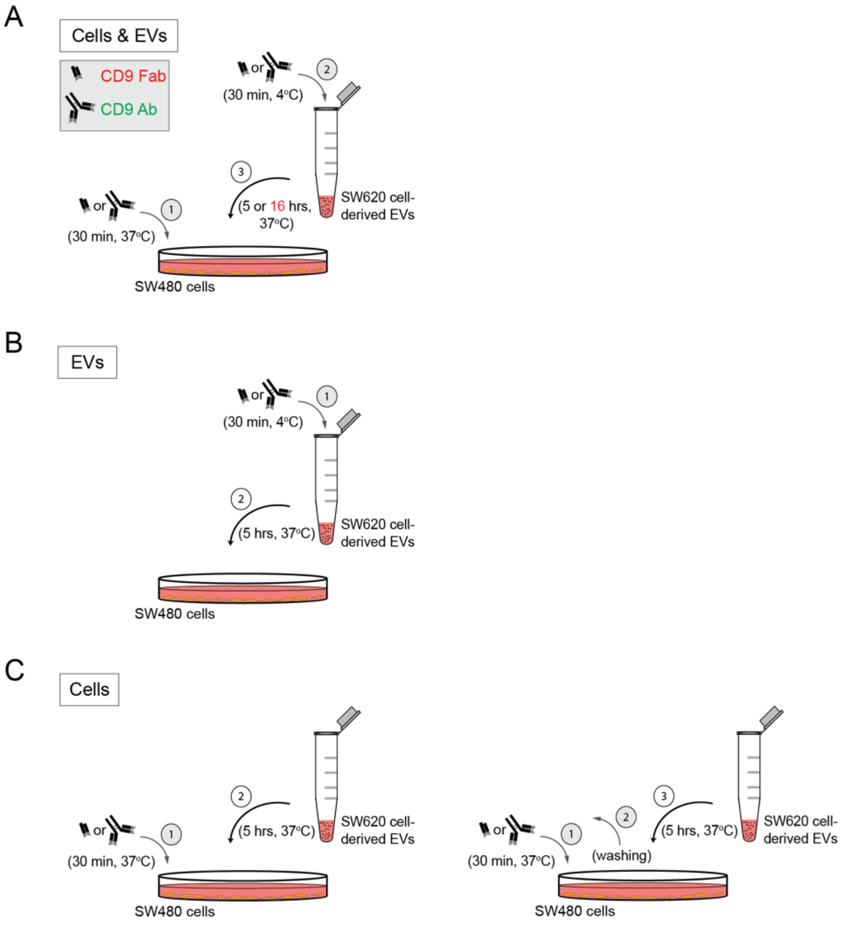

Figure S1. Schematic representation of the three main protocols used to study the impact of SW620 cell-derived on SW480 cells. **(A-C)** SW480 cells and SW620-cell-derived EVs were pre-incubated with either CD9 Fab or divalent Ab for 30 min at 37 °C or 4 °C, respectively, prior their co-incubation at 37 °C for 5 (or 16) h in the presence of Abs. This is referred to as protocol #1 (**A**). As alternatives, EVs or cells alone were pre-incubated with CD9 Fab or divalent CD9 Ab and then added to cells and EVs, respectively. These are referred to as protocol #2 (**B**) and protocol #3 (**C**), respectively. Note that in all conditions, Abs were not removed before co-incubation of cells and EVs, resulting in different Ab concentrations during the cell-EV incubation. As a control, Abs were omitted. Alternatively, Abs were removed from the cells after the 30-min pre-incubation and before the addition of EVs, which were not pre-incubated with Abs. This variant of protocol #3 is referred to as protocol #3' (**C**). Various experimental steps are numbered.

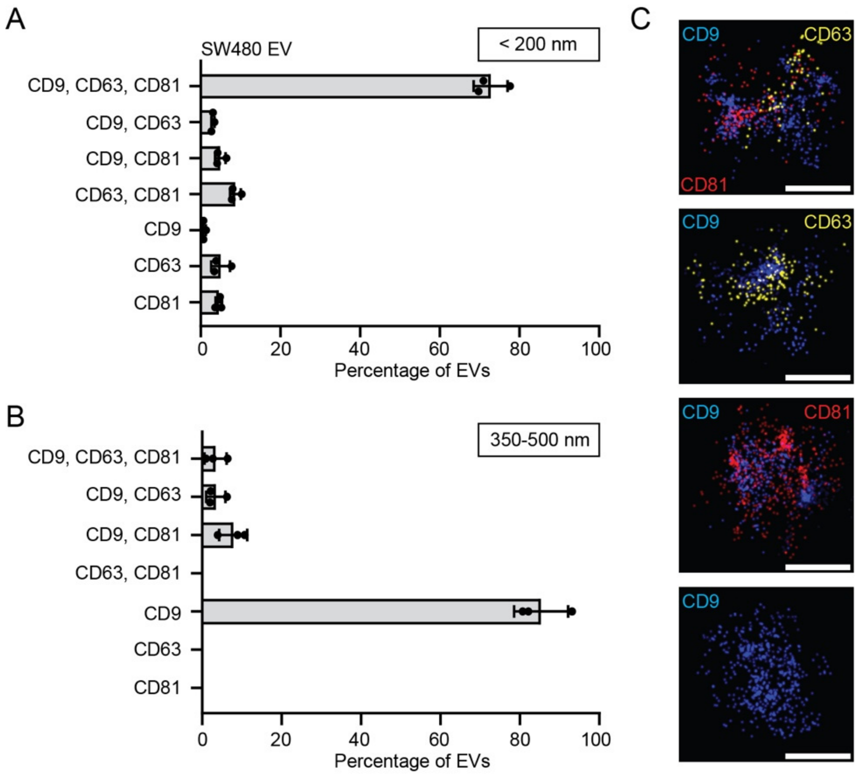

Figure S2. Characterization of small and large EVs released by SW480 cells. (**A-C**) Small (<200 nm, **A**) and large (350 to 500 nm, **B**) EVs derived from SW480 cells were imaged after immunolabeling of three tetraspanins as indicated using dSTORM, and the percentage of single, double and triple positive EVs was quantified. Means ± S.D. and individual values for three experiments are shown (n > 5000 (**A**) or 100 (**B**) EVs per experiment). Representative images of large EVs immunolabeled for CD9, CD63, and CD81 and pseudo-colored as indicated are shown (**C**). Note that the majority of large EVs are positive only for CD9. Scale bars, 200 nm.

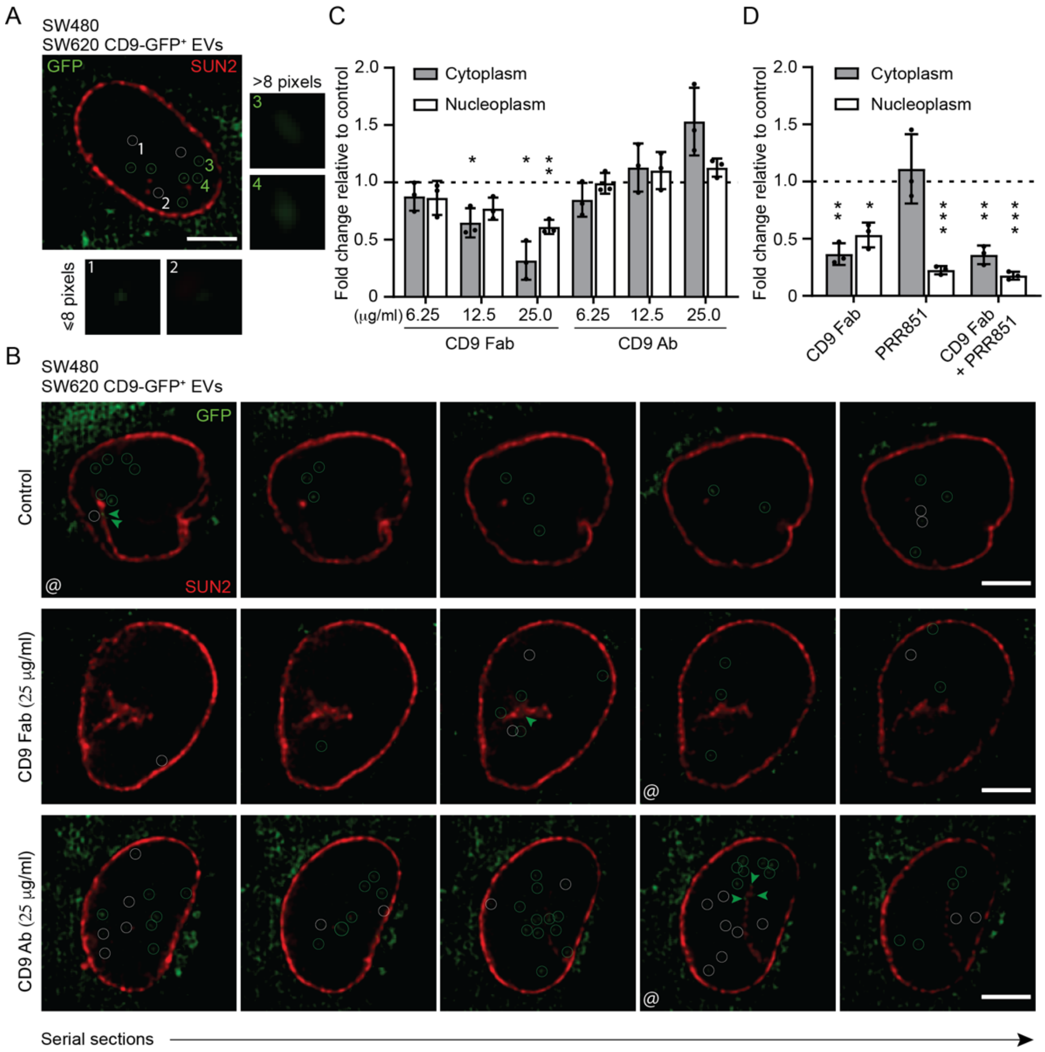

Figure S3. Quantification EV-derived CD9-GFP signal in the nucleoplasm of recipient SW480 cells. (**A**, **B**) SW480 cells exposed to fluorescent EVs (1×10^9 particles) derived from CD9-GFP⁺ SW620 cells were immunolabeled for SUN2 and analyzed by CLSM. To measure GFP fluorescence in the nucleoplasm, a serial x-y optical section through the cells was collected (20 sections, and one (**A**) or five (**B**) are displayed as examples), and each individual section (0.45 µm thick) was evaluated by analyzing ROIs delimitated by SUN2⁺ inner nuclear membrane labeling using the <<analyze function>> in Fiji. Because faint (auto)fluorescent signals equal to or less than 8 pixels (see enlargements 1 and 2, white circle) can be detected in cells not exposed to CD9-GFP⁺ EVs (data not shown), the threshold was set at a level greater than 8 pixels (#3 and 4, green circle) (**A**). Examples of serial sections of SW480 cells exposed to anti-CD9 Fab and divalent Ab or not (Control) as indicated before their 5-hour-co-incubation with CD9-GFP⁺ EVs, which themselves were exposed or not to CD9 Abs (**B**, for detail see Figure 3C). Arrowheads indicate CD9-GFP signal in nuclear envelope invaginations. Note that the images indicated by @ are also shown in Figure 3C. (**C**) The fold change of cytoplasmic and nuclear signals of CD9-GFP⁺ EVs of cells treated with anti-CD9 Fab or divalent Ab (original data are from Figure 3) relative to the control (dashed line) is shown. (**D**) SW480 cells pre-treated with PRR851 (10 µM) or without in the presence or absence of anti-CD9 Fab (25 µg/ml) for 30 min were incubated with EVs derived from CD9-GFP⁺ SW620 cells for 5 h. The fold change relative to the control (i.e., without Ab and drug, dashed line) was then measured as above. Means ± S.D. and individual values for each experiment are shown (n = 3). p values correspond to *, $p < 0.05$; **, $p < 0.01$; ***, $p < 0.001$. Scale bars, 5 µm.

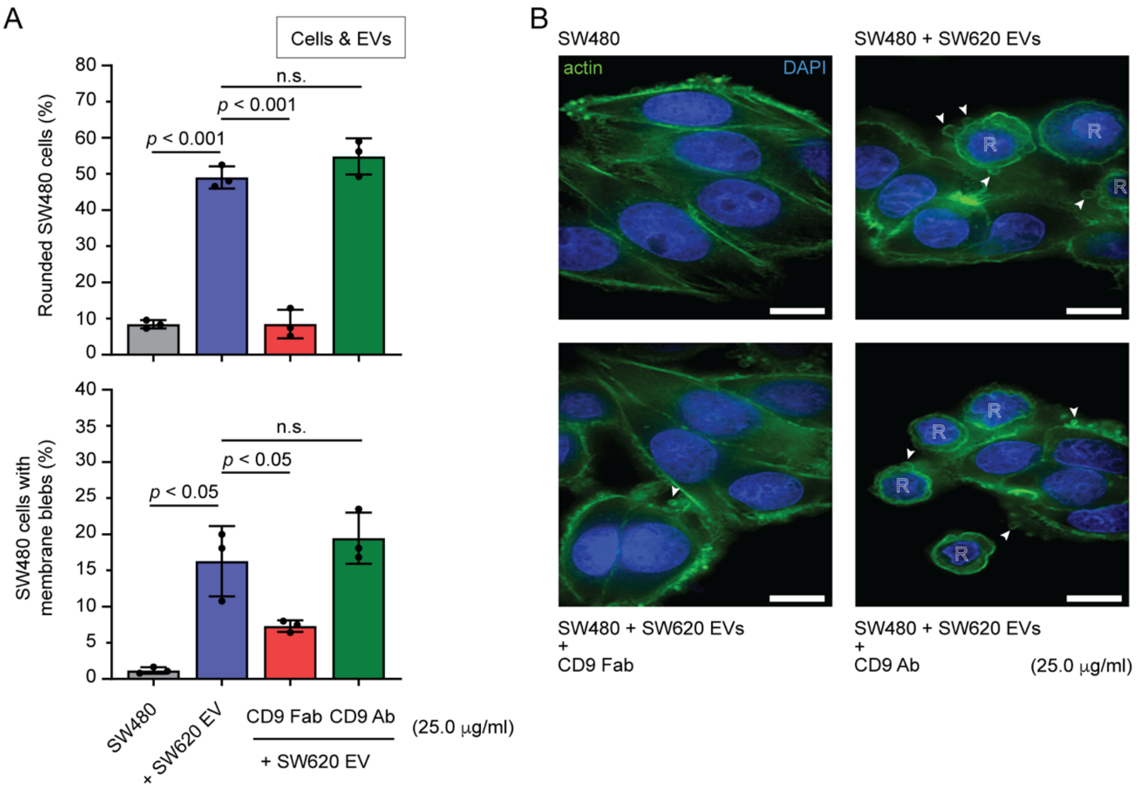

Figure S4. Effects of CD9 Fab and divalent Ab on the pro-metastatic morphological alterations of SW480 cells exposed to SW620 cell-derived EVs. (**A, B**) SW480 cells and SW620 cell-derived EVs (1 × 10^9 particles) were individually pre-incubated for 30 min with 25 μg/ml of anti-CD9 Fab or divalent Ab as indicated before their co-incubation for 16 h in the presence of Abs as described for protocol #1. As negative and positive controls, cells were not exposed (SW480) or were exposed to EVs (+ SW620 EV) in the absence of Ab, respectively. Afterward, fixed cells were stained with DAPI and fluorochrome-conjugated phalloidin to label nuclei and actin filaments, respectively, before observation by CLSM. The percentage of cells with rounded morphology (top panel) or membrane blebs (bottom panel) was quantified (**A**). Means ± S.D. and individual values for each experiment are shown (n = 3). At least 100 cells were evaluated per experiments. p values are indicated. n.s., not significant. Representative images were displayed for each condition (**B**). Single sections are presented. Rounded cell morphology and membrane blebs induced by EVs are indicated by R letter or arrowhead, respectively. Scale bars, 10 μm.

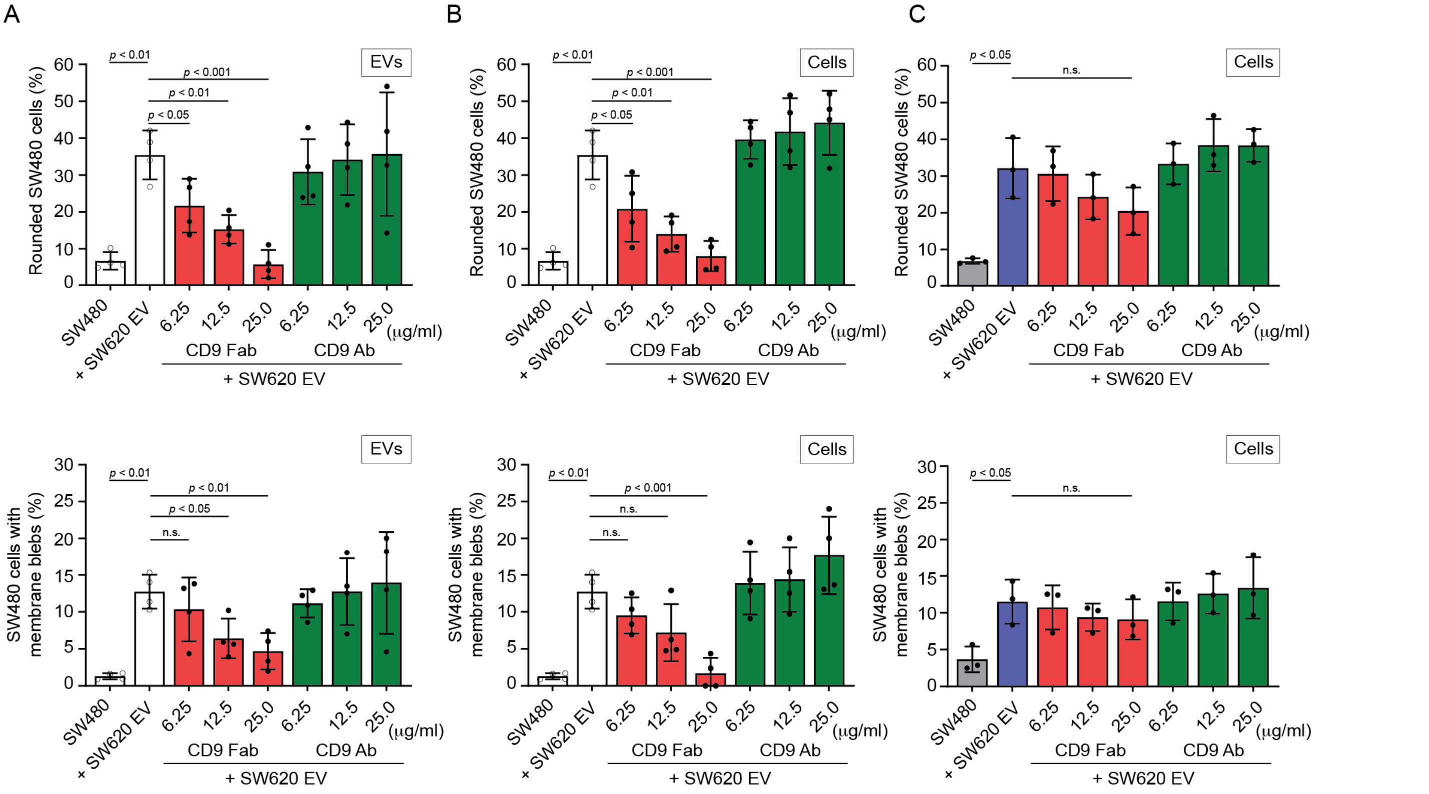

Figure S5. Effects of different concentrations of CD9 Fab and divalent Ab on the pro-metastatic morphological alterations of SW480 cells exposed to SW620 cell-derived EVs. (**A-C**) SW620 cell-derived EVs (**A**, 1×10^9 particles) or SW480 cells (B) were pre-incubated for 30 min with different concentrations of anti-CD9 Fab (red) or divalent (green) Ab as indicated prior to their co-incubation for 5 h with cells (**A**) or EVs (**B**), which were not exposed to Abs as described for protocols #2 and #3, respectively. In both protocols, Abs added for the pre-incubation were not removed. As negative and positive controls, cells were not exposed (SW480, grey) or were exposed to EVs in the absence of Ab (+ SW620 EV, blue). Controls (**A**, **B**, white) are displayed for comparison (for detail see Figure 4). Alternatively, Abs were removed from cells prior to the addition of EVs according to protocol #3′ (**C**). All cells were fixed and stained with DAPI and fluorochrome-conjugated phalloidin to label nuclei and actin filaments, respectively, before observation by CLSM. The percentage of cells harboring a rounded morphology (top panels) or membrane blebs (bottom panels) were assessed and graphed. The target of the pre-treatment with Abs is indicated (**A**, EVs; **B** and **C**, Cells). Means ± S.D. and individual values for each experiment are shown (n = 3-4). At least 100 cells were evaluated per experiments. p values are indicated. n.s., not significant.
